# Short‐term echocardiographic evaluation by global longitudinal strain in patients with heart failure treated with sacubitril/valsartan

**DOI:** 10.1002/ehf2.12656

**Published:** 2020-03-31

**Authors:** Simone Mazzetti, Chiara Scifo, Raffaele Abete, Davide Margonato, Margherita Chioffi, Jessica Rossi, Matteo Pisani, Giovanni Passafaro, Massimiliano Grillo, Daniele Poggio, Andrea Mortara

**Affiliations:** ^1^ Department of Clinical Cardiology Policlinico di Monza Via Amati, 111 20900 Monza MB Italy; ^2^ Department of Cardiology University of Pavia Pavia Italy

**Keywords:** Heart failure, Heart failure with reduced ejection fraction, Angiotensin receptor neprilysin inhibitor, Global longitudinal strain, Renin–angiotensin–aldosterone system, Neprilysin

## Abstract

**Aims:**

The angiotensin receptor neprilysin inhibitor (ARNI) sacubitril/valsartan reduces mortality and hospitalizations in patients with heart failure and reduced ejection fraction (HFrEF). Favourable effects on haemodynamic and functional parameters have been observed in patients with HFrEF undergoing ARNI therapy, using standard transthoracic echocardiography. Global longitudinal strain (GLS) assessment uses a semi‐automatic procedure to provide a reliable and repeatable method that improves the detection of early changes of contractile function. We aimed to assess the effects of ARNI on GLS and myocardial mechanics in patients with HFrEF.

**Methods and results:**

Thirty patients with New York Heart Association class II–III HFrEF were treated with ARNI and monitored using standard echocardiographic examination and GLS measurements at baseline, 3 months, and 6 months. ARNI therapy resulted in a significant reduction of ventricular volumes and a significant increase in left ventricular ejection fraction at 6 months but not 3 months by standard transthoracic echocardiography (left ventricular ejection fraction from 28 ± 8% at baseline to 34 ± 12% at 6 months, *P* < 0.001). Non‐significant differences in the size of the left atrium, right ventricular function, and pulmonary pressures were found at 6 months. By using GLS, there was a progressive improvement of all strain parameters by 3 months. The improvement showed a progressive trend over time and maintained significance at 6 months: GLS 4ch −7.2 ± 4.8% at baseline vs. −7.5 ± 3.9% at 3 months (*P* = 0.025) and − 9.2 ± 5.2% at 6 months (*P* = 0.0001); AVG GLS −6.9 ± 4.3 at baseline vs. −7.9 ± 4.2 at 3 months (*P* = 0.04) and − 8.8 ± 4.4 at 6 months (*P* = 0.035); GLS endo 8.2 ± 4.8 at baseline vs. −9.0 ± 4.8 at 3 months (*P* = 0.05) and − 10.1 ± 5.1 at 6 months (*P* = 0.001).

**Conclusions:**

Sacubitril/valsartan induces an early benefit on left ventricular remodelling, which is captured by myocardial strain and not by standard echocardiography. Strain method represents a practical tool to assess early and minimal variations of left ventricular systolic function.

## 1 Introduction

In the PARADIGM‐HF trial, combination therapy with sacubitril/valsartan, the first‐in‐class ARNI, showed relevant results in terms of reduction of both mortality and hospitalizations together with an improvement in the quality of life in patients with heart failure and reduced ejection fraction (HFrEF).^1^ More recently, a meta‐analysis of 21 randomized controlled trials in a total of 69229 patients compared the relative efficacy of renin–angiotensin–aldosterone system blockers for HFrEF.^2^ Angiotensin receptor neprilysin inhibitor (ARNI) had the highest probability of reducing the risk of all‐cause mortality and preventing hospitalization for heart failure, compared with angiotensin‐converting enzyme inhibitors (ACEIs), angiotensin receptor blockers, and aldosterone receptor antagonists, alone or in combination.^2^ Recent studies have shown that ARNI led to a greater reduction in N‐terminal pro B‐type natriuretic peptide (NT‐proBNP) than enalapril among patients admitted with acute decompensated heart failure.^3^, ^4^, ^5^ The reduction in NT‐proBNP achieved with ARNI was also correlated with signs of reverse cardiac remodelling at 1 year, in terms of an increase in left ventricular ejection fraction (LVEF) and a decrease in indexed left ventricular end‐diastolic and systolic volumes.^3^, ^6^ ARNI also significantly improved cardiac volumes and ejection fraction, with standard transthoracic echocardiography (TTE), and improvements in mitral regurgitation and diastolic function parameters were also observed, with a medium‐term dose‐dependent effect.^7^, ^8^

However, it is known that evaluation by standard TTE is limited by intra‐observer variability. Global longitudinal strain (GLS) assessment, on the other hand, through a semi‐automatic procedure that identifies the endocardial border and its movement over time, appears to have more sensitivity and specificity in the detection of left ventricular systolic dysfunction, thus improving the detection of early changes of contractile function, in contrast with standard biplane ejection fraction evaluation.^9^, ^10^, ^11^, ^12^ The aim of our study was to assess the effects of ARNI on GLS and myocardial mechanics in patients with HFrEF.

## 2 Methods

### 2.1 Study population

Patients referred to our heart failure outpatient department who were in New York Heart Association (NYHA) class II–III and with ejection fraction <40%, provided that they were on optimized medical treatment (OMT) since at least 6 months and eligible for ARNI, were screened for enrolment, regardless of heart failure aetiology. Of the 45 patients initially screened, 15 were excluded because of the presence of conditions limiting GLS analysis: atrial fibrillation with extreme irregular RR interval, or frequent or repetitive supraventricular or ventricular ectopic beats (eight patients), or a poor echocardiographic window (seven patients). The remaining 30 patients (nine women) with a mean age of 64 ± 10.7 years and body mass index 3.2 ± 2.5 kg/m^2^, were enrolled for clinical and instrumental evaluation.

The study was approved by the local Ethics Committee in accordance with the Declaration of Helsinki, and all patients signed informed consent before participation in the study. For each patient, baseline echocardiographic examination performed in the previous 3 to 6 months was considered as a baseline (pre‐treatment) evaluation. For all enrolled patients, before starting ARNI, outpatient cardiologic examination was performed with clinical visit, physical measurements of vital signs (systolic arterial pressure, diastolic arterial pressure, pulse rate, and weight), body mass index calculation, 12‐lead electrocardiogram; blood tests inclusive of complete blood count, renal function, electrolytes, and BNP/NT‐proBNP were recorded; concomitant therapy was registered; the ARNI was started with a dose compatible with the patient's clinical history and co‐morbidities, according to specific prescription criteria, and ambulatory follow‐up was scheduled.

### 2.2 Follow‐up management

Starting from baseline (pre‐treatment), for all patients, follow‐up at 3 and 6 months was scheduled, including clinical examinations, blood tests, and TTE. All subjects were alive at follow‐up. During the period of observation, there were six rehospitalizations, only four of which were for acute decompensated heart failure, with three of these relating to a single patient.

### 2.3 Echocardiographic measurements

All patients underwent a standard echocardiographic examination with the same Vivid 9 Digital Ultrasound System echocardiograph (GE Medical Systems, Chicago, Illinois, United States). All echocardiographic tests were performed by the same sonographer. Three cardiac cycles in cine loop format were recorded for offline analysis. Left atrial and ventricular volumes were measured according to the recommendations of the American Society of Echocardiography.^13^ LVEF was assessed with Simpson's biplane method by two blinded qualified operators (three measures for each projection; the average value was recorded). The diastolic function was evaluated by the analysis of transmitral flow (E/A, E/E′, and deceleration time). Any valvular diseases were evaluated according to the American Society of Echocardiography guidelines,^14^ with particular emphasis on functional mitral valve regurgitation. Pulmonary pressures were estimated by tricuspid regurgitation peak and inferior vena cava diameter. Baseline measurements were repeated at 3 and 6 months by the same operators with the same digital ultrasound system.

### 2.4 Global longitudinal strain measurements

Left ventricle (in apical four‐chamber, two‐chamber, and three‐chamber views) was divided into six segments, each of which was assessed using a dedicated software (EchoPac Dimension 06; GE Healthcare, Chicago, Illinois, United States) for the 2D S/SR analysis, which allows the evaluation of the deformation and the rate of myocardial systolic deformation starting from the standard two‐dimensional acquisitions. By tracing the endocardial contour on a telediastolic frame, the software automatically calculates the endocardial contour in subsequent frames using a speckle tracking analysis. The adequacy of the tracking can be verified in real time, making corrections by changing the regions of interest or changing the contour manually.

Furthermore, the depth of the region of interest (thickness of the endocardial and epicardial pixels that allows the software to perform beat to beat analysis of the strain) can be adjusted.

The systolic values of the S/SR peak were calculated in all the subjects, and the GLS value was recorded in the three main projections (apical 4ch, 2ch, and 3ch) and the average GLS value and the corresponding GLS value of the endocardial layer and the epicardial layer (GLS endo and GLS epi, respectively).

To simplify the analysis, for each patient, the values of the average segmental strain and the average segmental strain rate obtained from the arithmetic mean of the values of the six segments under examination were also calculated. Two blinded operators analysed all the data from the echocardiographic examination.

### 2.5 Statistical analysis

Data were expressed as means ± standard deviation. Comparisons between subjects were performed using the Wilcoxon‐signed rank test. A value of *P* < 0.05 was considered statistically significant. The data were analysed using SPSS 10.0 software (SPSS Inc., Chicago, IL, USA).

## 3 Results

The main clinical characteristics of the 30 patients enrolled in the study are summarized in *Table* 1. At baseline, 26 patients (86.6%) were treated with an ACEI (six with enalapril at an average dosage of 31.7 ± 9.8 mg/day, 11 with ramipril at an average dosage of 5.4 ± 2, 8 mg/day, and nine with captopril at an average dosage of 20.3 ± 12 mg/day); four patients (13%) with an angiotensin receptor blocker (three with valsartan at an average dosage of 20.4 ± 12 mg/day and one with candesartan at a dose of 32 mg/day).

**Table 1:** Patients characteristics at baseline and after 3 and 6 months of follow‐up (*n* = 30)

	Baseline	3 months	6 months
Age (years), mean ± SD	64 ± 10.7		
Female, pt (%)	9 (30)		
Mean BMI, kg/m^2^	32 ± 2.55		
Haemodynamic parameters, mean ± SD			
Ejection fraction	28 ± 8	28 ± 9	34 ± 12
SBP, mmHg	121.11 ± 17	119.40 ± 19.2	113.6 ± 20.2
DBP, mmHg	76.85 ± 15.3	75.36 ± 12.50	72.52 ± 13.4
MBP, mmHg	91.67 ± 13.1	90.4 ± 13.3	86.12 ± 11.7
Pulse rate, b.p.m.	66.33 ± 25.51	66.33 ± 11	66.26 ± 9
Blood test parameters			
Creatinine (mg/dL), mean ± SD	1.26 ± 0.47	1.30 ± 0.62	1.67 ± 0.75
Aetiology, pt (%)			
Ischaemic	12 (40.0)		
Idiopathic	16 (53.3)		
Valvular	2 (6.67)		
NYHA II	16 (53.3)		
NYHA III	14 (46.67)		
Co‐morbidities, pt (%)			
Diabetes	6 (20.0)		
BP	2 (6.67)		
CKD (eGFR <40 mL/min/1.73 m^2^)	5 (16.7)		
Anaemia (Hb <12 g/dL)	1 (3.33)		
Obesity (BMI >30)	4 (13.3)		
Device, pt (%)			
CRT	6 (20.0)		
ICD	16 (53.3)		
No electrical therapy	8 (26.67)		

BMI, body mass index; BP, blood pressure; CKD, chronic kidney disease; CRT, cardiac resynchronization therapy; eGFR, estimated glomerular filtration rate; Hb, haemoglobin; ICD, implantable cardioverter defibrillator; NYHA, New York Heart Association; pt, patient; SD, standard deviation.

All patients were on diuretic treatment (furosemide in 100% of cases; average dose, 62.07 ± 49.38 mg/day), 20 patients (66.7%) were receiving mineralocorticoid receptor antagonist therapy at an average dosage of 40.05 ± 29.62 mg/day, 28/30 patients were on beta‐blocking therapy (ninety‐nine with bisoprolol at an average dosage of 3.68 ± 2.2 mg/day, one with metoprolol at 50 mg/day, and eight with carvedilol at an average dosage of 19.3 ± 15.3 mg/day).

### 3.1 Angiotensin receptor neprilysin inhibitor at baseline and up‐titration regimens

After the baseline visit, ARNI was started at the maximum dosage (97/103 mg bid) in two patients (6.7%) who had systolic blood pressure values >130 mmHg and had received previous treatment with captopril 50 mg tid. In 13 patients (43.3%), ARNI was started at an intermediate dosage (49/51 mg bid); in the remaining 15 (50%), the treatment was started at a low dosage (24/26 mg bid).

**Figure 1:**
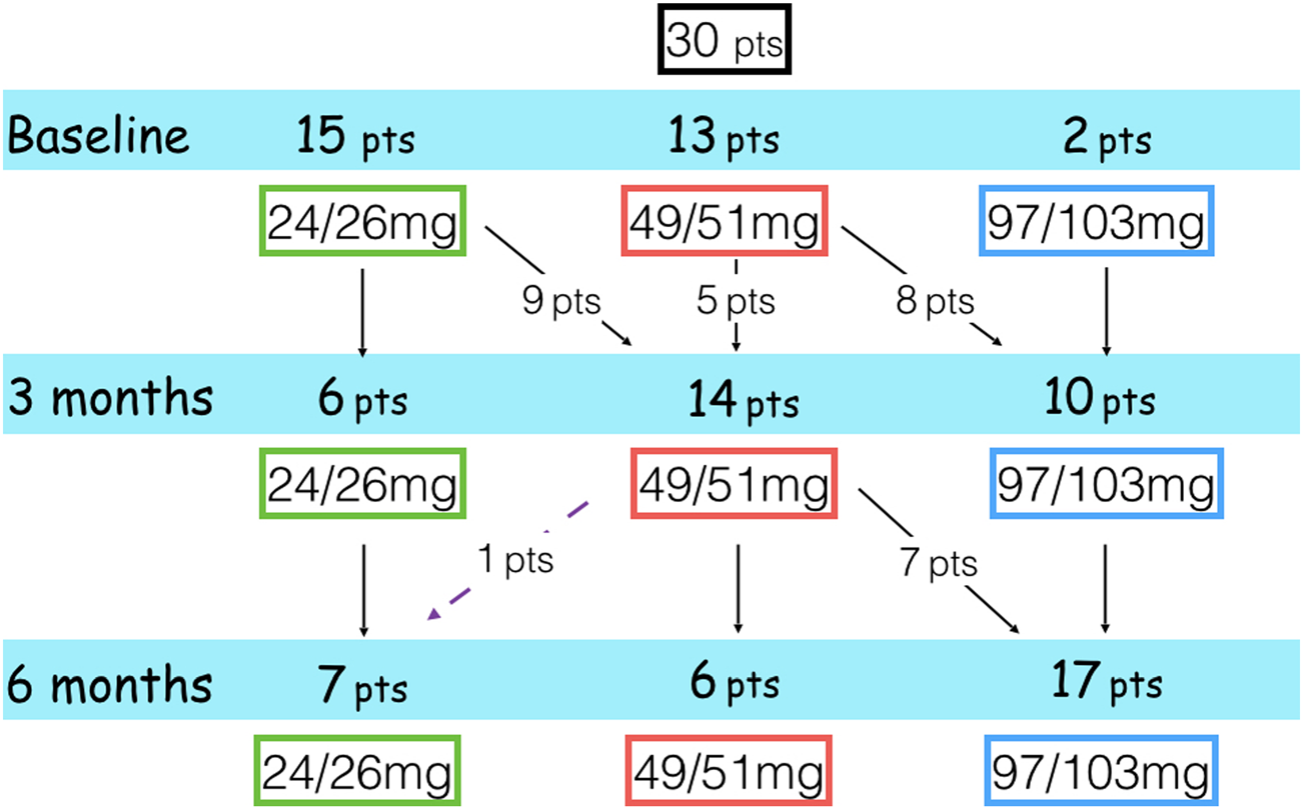
Flow chart of up‐titration of angiotensin receptor neprilysin inhibitor during treatment. Pts, patients.

**Figure 2:**
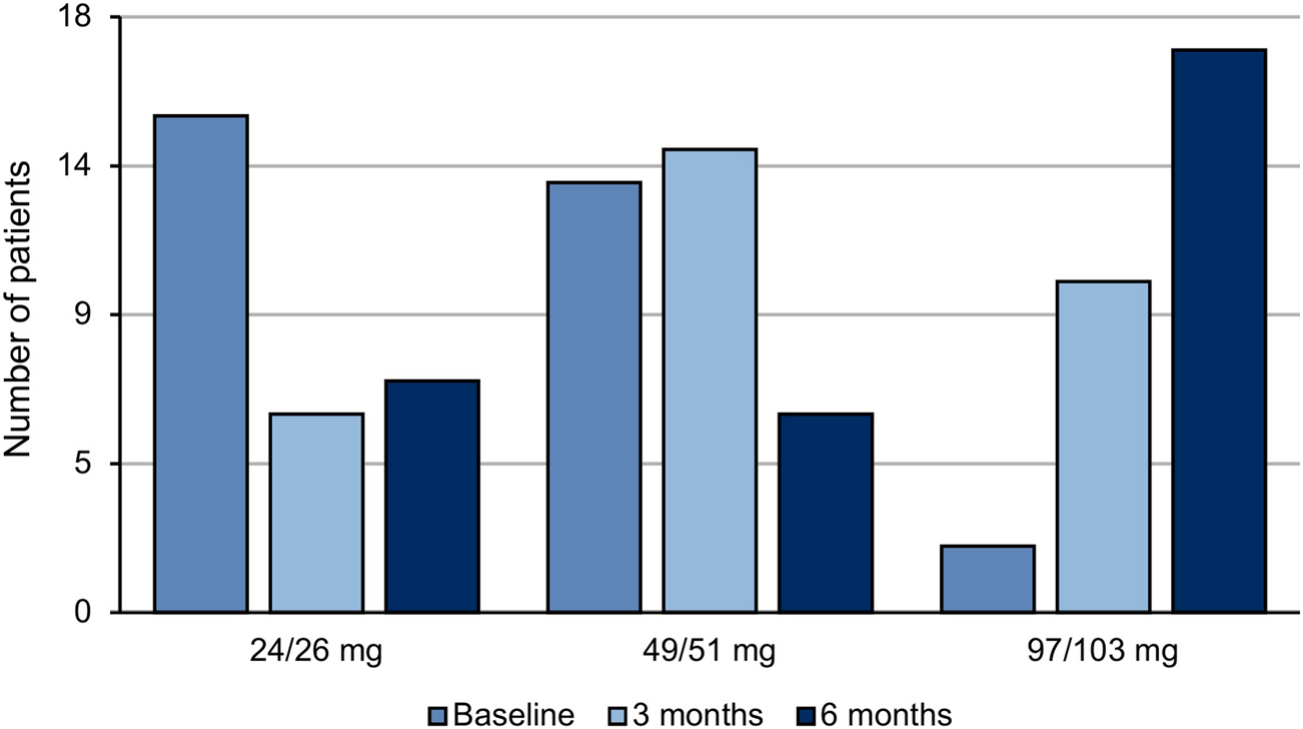
Angiotensin receptor neprilysin inhibitor dosage during follow‐up. Baseline (blue), after 3 months of follow‐up (light blue), and after 6 months of follow‐up (dark blue).

Over the period of study, slow titration of ARNI was carried out (*Figures* *1* and *2*), compatible with the haemodynamic conditions and the haemato‐chemical data. At 3 months, it was not possible to carry out up‐titration in 13 patients (46.4%): two patients were already at maximal dosage at baseline, eight patients because of hypotension (systolic blood pressure < 110 mmHg), one patient because of evidence of advanced chronic kidney disease at the time of two serial controls (estimated glomerular filtration rate < 30 mL/min/1.73 m^2^), in one case for gastrointestinal intolerance (sense of nausea and dysentery) and in the last case due to manifest lack of compliance.

At 6 months, 17/30 patients (56.7%) reached the maximum dosage of ARNI (97/103 mg bid), while it was not possible to proceed with titration in 12 patients (eight cases because of hypotension and four because of worsening of renal function). In one patient, it was necessary to reduce the dosage due to a reduced glomerular filtrate. At the end of the titration phase, 17 patients received the maximum dosage of ARNI, six patients the intermediate dosage, and seven patients the minimum one (see *Figures* *1* and *2*).

### 3.2 Effect of angiotensin receptor neprilysin inhibitor on clinical parameters

During the 6 months of follow‐up, a slight reduction in mean blood pressure values was observed at 3 months, with a further reduction at 6 months (baseline 91.67 ± 13.1 mmHg, 90.4 ± 13.3 mmHg at 3 months, and 86.12 ± 11.7 mmHg at 6 months) (*Table* 1). Regarding blood chemistry values, an increase in mean creatinine level was noted (from a mean of 1.26 ± 0.47 mg/dL at baseline to 1.67 ± 0.75 at 6 months; *P* < 0.02), and there was a non‐linear trend in reduction of NT‐proBNP values (from 1496 pg/dL at baseline to 1014 pg/dL at 6 months). Non‐significant variations of potassium levels were recorded. Finally, a substantial stability of the NYHA functional class was documented, and no change in the heart rate trend was calculated during the outpatient visit.

### 3.3 Echocardiographic parameters

Echocardiographic data are described in *Table* 2. Therapy with ARNI resulted in a significant reduction of ventricular volumes and a significant increase in LVEF at 6 months compared with baseline but not at 3 months. No significant differences were found at 6 months with regard to the size of the left atrium, right ventricular function (assessed by tricuspid annular plane systolic excursion), and pulmonary pressures. Even without achieving statistical significance, an improvement trend of the main diastolic function indices (E/A and deceleration time) was clearly observed.

**Table 2:** Echocardiographic parameters at baseline and after 3 and 6 months of follow‐up

	Baseline	3 months	6 months
LVEDV (mL)	178.36 ± 64.15	172.12 ± 72.30	163.08 ± 85.22#, *
LVESV (mL)	128.04 ± 53.29	123.76 ± 56.83	116.21 ± 75.31#, *
LVEF (%)	28 ± 8	28 ± 9	34 ± 12#, *
TAPSE (mm)	17 ± 4	17 ± 5	17 ± 7
LAV (mL)	84.3 ± 42	83 ± 47	85 ± 48
PAP (mmHg)	34.80 ± 9.28	30.60 ± 11.04	30.95 ± 17.02
E/A ratio	1.52 ± 1.05	1.26 ± 0.89	1.16 ± 1
DecT (s)	186.72 ± 82.64	238.65 ± 115.89	240.83 ± 133.18

DecT, deceleration time; E/A ratio, ratio between blood flow in early diastole (E wave) to flow in late diastole by atrial contraction (A wave); LVEDV, left ventricular end‐diastolic volume; LVESV, left ventricular end systolic volume; LAV, left atrial volume; LVEF, left ventricular ejection fraction; PAP, pulmonary artery pressure; TAPSE, tricuspid annular plane systolic excursion.

Values are mean ± standard deviation.

# *P* < 0.001 vs. baseline.

* *P* < 0.05 vs. 3 months.

### 3.4 Global longitudinal strain data

**Figure 3:**
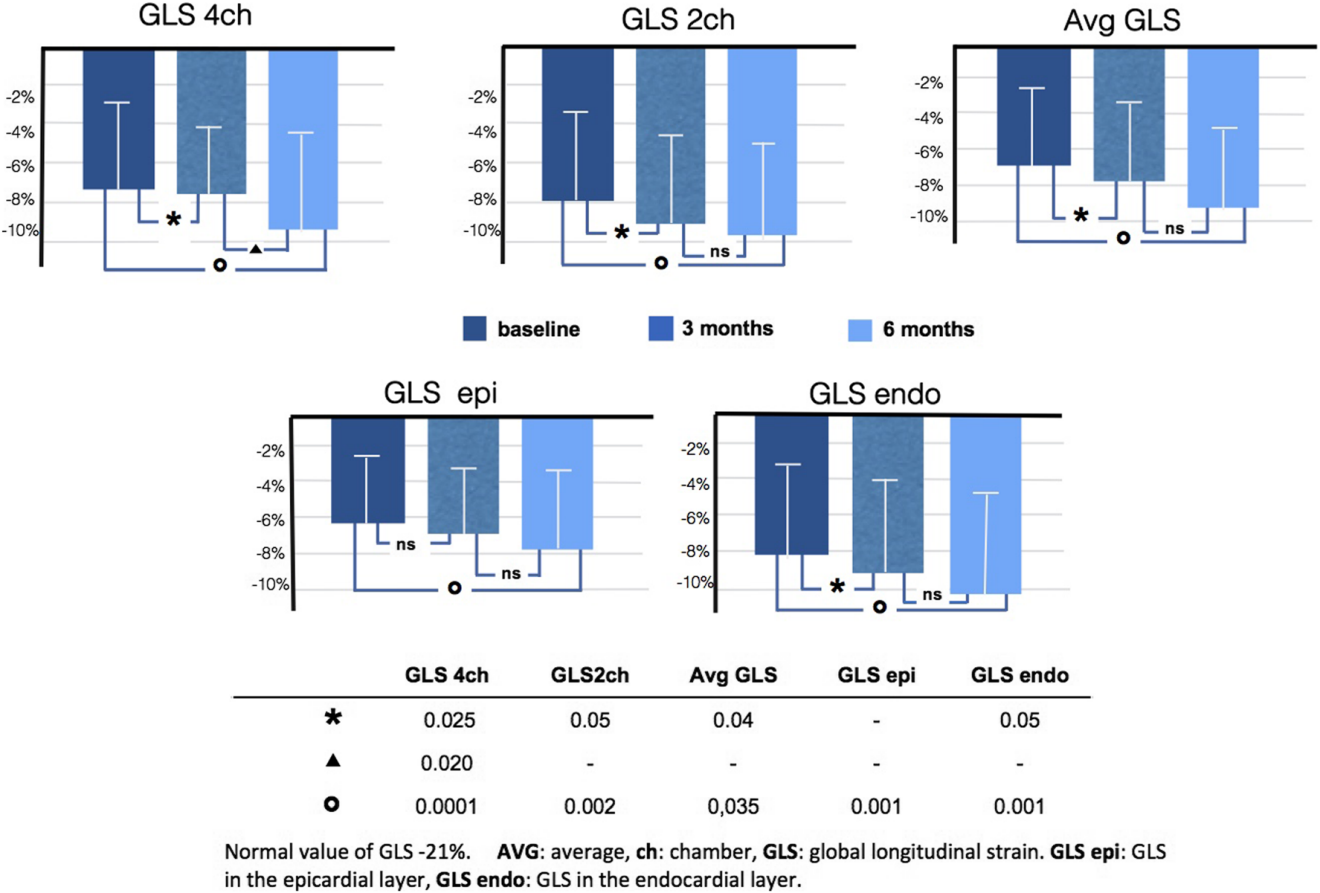
Global longitudinal strain at baseline and after 3 and 6 months of follow‐up.

**Figure 4:**
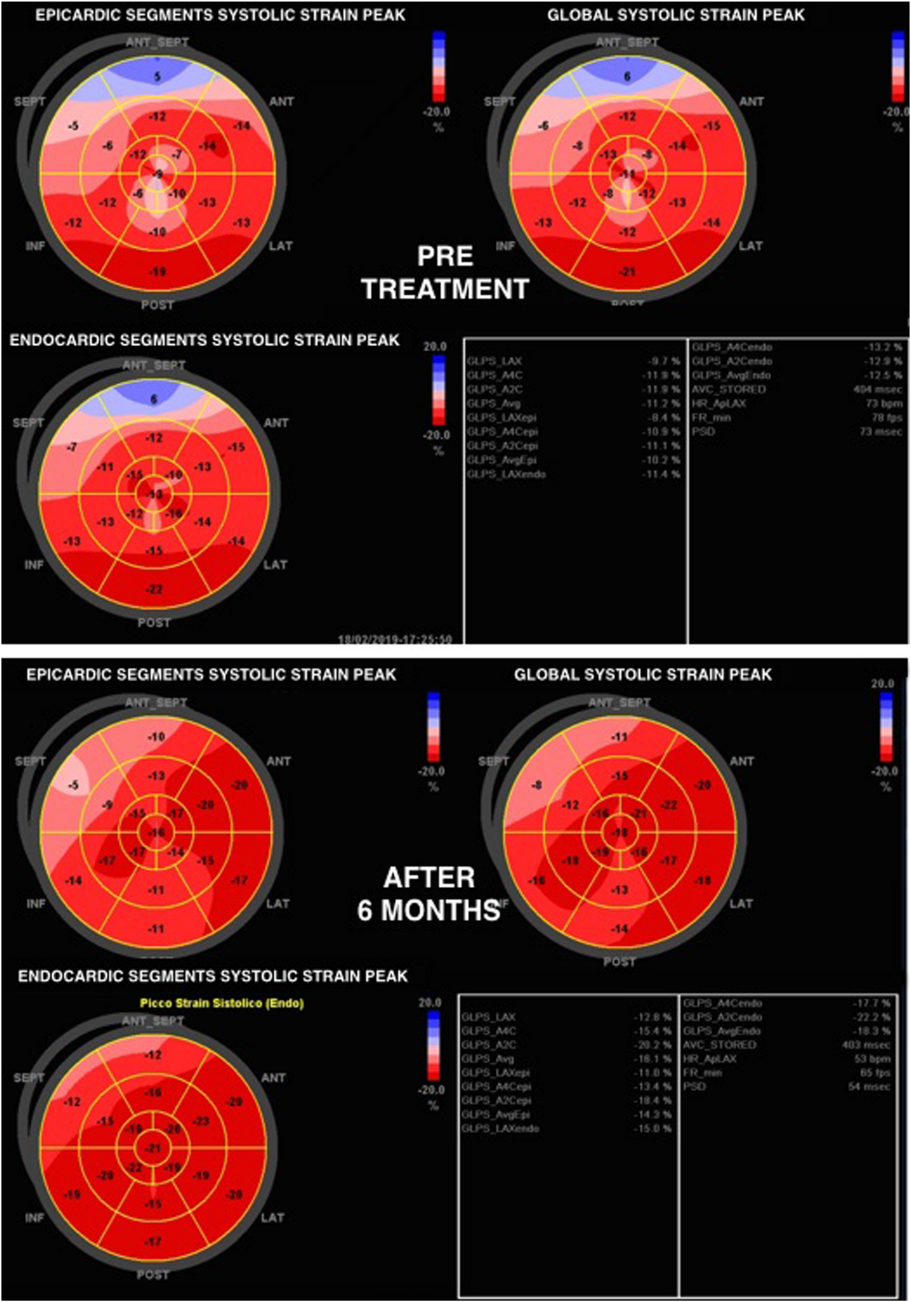
Example of evaluation of the systolic peak of strain on 16 segments model at baseline (pre‐treatment) and after 6 months of angiotensin receptor neprilysin inhibitor therapy.

The data related to the GLS in the two main projections (GLS 4ch and GLS 2ch), the value of the average GLS, and the corresponding value related to the endocardium layer and the epicardium layer (GS endo/GS epi, respectively) are shown in *Figure* 3. There is a progressive improvement of all strain parameters that becomes statistically significant already at 3 months for most of the parameters considered. This improvement maintained statistical significance even at 6 months, showing a progressive trend over time. An example of the evaluation of systolic peak of strain using the 16 segments model at baseline and after 6 months of ARNI is shown in *Figure* 4.

## 4 Discussion

This study shows that GLS is a sensitive method for the early assessment of the effects of pharmacological treatments, such as sacubitril/valsartan, on positive left ventricular remodelling. In fact, the improvement in GLS was statistically significant at 3 months, whereas for the standard ultrasound data, this statistical result was reached only after 6 months of follow‐up. Sacubitril/valsartan has been shown to be a well‐tolerated drug in the population with HFrEF, and it was possible to titrate the ARNI to the maximum dose (97/103 mg bid) in 69% of patients (17/30).

As noted in previous studies, therapy with ARNI produced a progressive lowering of the systolic–diastolic pressure values even in the short term,^15^, ^16^ with a significant reduction of the average arterial pressure at 6 months. In eight cases, there was a reduction of pressure that prevented complete up‐titration, although in the absence of specific symptoms or major episodes. In only one case, there was a marked deterioration of renal function, despite adjustment and modulation of diuretic therapy, which required a reduction of the dosage of ARNI; in the rest of the population, therapy with sacubitril/valsartan was well tolerated even in the presence of moderate‐to‐severe renal dysfunction at baseline.

The recording of echocardiographic values enabled a tendency towards an improvement in the main parameters of systolic function (telediastolic volume/telesystolic volume/LVEF) and a clear trend in the direction of progressive improvement of the diastolic function parameters to be recognized.

These data confirm previous data regarding the favourable remodelling observed in patients undergoing treatment with ARNI.^3^, ^6^, ^7^, ^8^ The particularity of this work was, however, that of adding the analysis of the GLS to the evaluation of standard echocardiography. In fact, this method provides independent and incremental prognostic information in addition to standard echocardiographic parameters in a series of clinical scenarios. Improvements of GLS after initiation of specific treatments have been demonstrated in hypertensive heart disease, in obesity, and in metabolic syndrome following treatment with spironolactone.^17^ However, many studies are still underway to assess whether changes in GLS values are associated with changes relevant to clinical management. Some progress has been made in the field of cardio‐oncology, where current guidelines for cardiotoxicity assessment related to chemotherapy support variations in GLS values from baseline of more reliable clinical predictive value than an absolute cut‐off.^18^ What is clear is that, with the availability of post‐processing algorithms, the assessment of GLS does not require a particularly long learning curve and should, therefore, become part of the technical and cultural background of the clinical cardiologist and become routine in the follow‐up of the ambulatory patient.^19^, ^20^ However, for the adoption of new technologies, as is the case with new therapies, education and training must be used to overcome initial inertia.

The previous intervendor variability in strain measurements represented a significant obstacle to the adoption of this method because it prevented the adoption of a standardized normal interval, and the use of different machines from one vendor to another could be a confusing element masking any differences present. Although the use of the echocardiography device of a single vendor is still advisable, the variability between different vendors has long been improved, allowing a comparable estimate of GLS regardless of the echocardiographic technology used.

Measurement of the systolic function by the volumetric method, while remaining a cornerstone in the nosological framework for risk assessment and management of all patients with heart disease is, however, affected by extreme interobserver variability. GLS improves the detection of systolic dysfunction, placing itself ‘beyond’ the LVEF. In particular, it has proven to be a very useful method in all the scenarios in which diastolic dysfunction represented a preponderant element. Furthermore, the semi‐automatic method of identifying the endocardial border and its movement over time makes it a reliable and repeatable method.

The measurement of the variation of GLS over time in our study cohort has allowed us to more promptly perceive small but significant improvements in the left ventricular function after starting therapy with ARNI. In fact, GLS significantly improved at 3 months at a stage when the LVEF and volumes, although in an improving trend, show only slight variations. The use of strain imaging, therefore, raises issues related to its adoption not only as an integral part in the staging and typing of HF but also as a tool for a better detailed phenotyping and a better risk assessment, providing a favourable impact on main cardiovascular outcomes.

Despite the encouraging results described, there are several limitations to the present study: observational study design, small sample size, and the lack of a control group do not allow a direct comparison between the effects of ARNI and OMT on left ventricular systolic function. However, all enrolled patients were in stable clinical status on OMT treatment since at least 6 months prior to starting ARNI, and all patients with a recent diagnosis of HF (i.e. <6 months) were excluded from the study (no naive patients). Therefore, all patients in our study had received OMT for a minimum of 6 months before starting ARNI, without any improvement in echo parameter, which suggests, as a consequence, that we can assume that all improvements seen in ventricular remodelling and in GLS analysis are likely to be attributable to the introduction of ARNI. Furthermore, our analysis of GLS at three time points specifically addresses the above concerns, and its results suggest that the improvements seen in GLS are very likely due to ARNI added to OTM. However, we cannot rule out a slight Hawthorne effect on our cohort of patients.

In conclusion, this study confirms the favourable effects of ARNI on left ventricular function and underlines how the strain method may today represent a practical tool for the capture of early and even minimal variations of left ventricular systolic function that would be less visible with the standard ejection fraction calculation. These encouraging results require confirmation in larger prospective cohorts with longer follow‐up.

## Conflict of interest

None declared.

## Author contributions

All authors were involved in the conception and design of the study and drafting of the manuscript, as well as revising the manuscript critically. All authors provided approval of the final version of the manuscript.

## Funding

This work did not receive any specific grant from funding agencies in the public, commercial, or not‐for‐profit sectors.
